# A Tightly Controlled Conditional Knockdown System Using the *Tol2* Transposon-Mediated Technique

**DOI:** 10.1371/journal.pone.0033380

**Published:** 2012-03-13

**Authors:** Tokuichi Iguchi, Hideshi Yagi, Chen-Chi Wang, Makoto Sato

**Affiliations:** 1 Division of Cell Biology and Neuroscience, Department of Morphological and Physiological Sciences, Faculty of Medical Sciences, University of Fukui, Fukui, Japan; 2 Research and Education Program for Life Science, University of Fukui, Fukui, Japan; 3 Faculty of Medical Sciences, Child Development Research Center, University of Fukui, Fukui, Japan; Institut de la Vision, France

## Abstract

**Background:**

Gene knockdown analyses using the *in utero* electroporation method have helped reveal functional aspects of genes of interest in cortical development. However, the application of this method to analyses in later stages of brain development or in the adult brain is still difficult because the amount of injected plasmids in a cell decreases along with development due to dilution by cell proliferation and the degradation of the plasmids. Furthermore, it is difficult to exclude the influence of earlier knockdown effects.

**Methodology/Principal Findings:**

We developed a tightly controlled conditional knockdown system using a newly constructed vector, pT2K-TBI-shRNAmir, based on a *Tol2* transposon-mediated gene transfer methodology with the tetracycline-inducible gene expression technique, which allows us to maintain a transgene for a long period of time and induce the knockdown of the gene of interest. We showed that expression of the endogenous amyloid precursor protein (APP) was sharply decreased by our inducible, stably integrated knockdown system in PC12 cells. Moreover, we induced an acute insufficiency of Dab1 with our system and observed that radial migration was impaired in the developing cerebral cortex. Such inhibitory effects on radial migration were not observed without induction, indicating that our system tightly controlled the knockdown, without any expression leakage *in vivo*.

**Conclusions/Significance:**

Our system enables us to investigate the brain at any of the later stages of development or in the adult by utilizing a knockdown technique with the aid of the *in utero* electroporation gene transfer methodology. Furthermore, we can perform knockdown analyses free from the influence of undesired earlier knockdown effects.

## Introduction

During the development of the cerebral cortex, neurons are generated in the ventricular zone (VZ) and then migrate outward to the cortical plate (CP), an event that is called “radial migration.” These neurons stop their movement and settle into the six layers of the cortex in an inside-out pattern, whereby early-born neurons are positioned in the deeper layers, and later-born neurons are located in the more superficial layers, migrating outward by passing the earlier born neurons [1].

In the later stage of the cortical development, just after radial migration ends, neurons that have settled in the cortex continue to elongate their axons toward their targets. In mice, layer-specific stereotyped projection patterns are established for each layer of the cortex: layer V neurons, which are born around embryonic day (E)12.5, mainly project to the subcortical regions as the subcortical projection, whereas layer II/III pyramidal neurons, which are born around E14.5, mainly project to the contralateral cortex as the callosal projection [2], [3].

In such studies of cortical development, *in utero* electroporation gene transfer is frequently employed because it enables us to perturb the *in vivo* gene expression in a convenient manner compared to conventional techniques, such as making genetically modified animals [4], [5]. Using the *in utero* electroporation gene transfer, it has been revealed that various molecules are involved in the development of the cerebral cortex by controlling radial migration. One such example is an unexpected activity of the amyloid precursor protein (APP), for which involvement in Alzheimer's disease has been established, in radial migration by controlling Disabled-1 (Dab1) activity downstream [6]. Dab1 is an adapter protein that is crucial for the signal transduction of Reelin signaling, which regulates neuronal migration during corticogenesis. A disruption in *Dab1* results in the malformation of the cerebral cortex in which lamination is inverted such that neurons do not keep to the inside-out pattern [7], [8].

Although *in utero* electroporation gene transfer is a powerful tool for studying the events in early corticogenesis, there are some difficulties in applying this technique to the later stages of development, such as the formation of a neuronal network, which includes axon path-finding and synaptogenesis, due to the following reasons: (1) Gene expression from the injected plasmids decreases along with development due to dilution by cell proliferation and the degradation of the plasmids. (2) It is difficult to exclude the influence of earlier gene manipulation. For example, modified neuronal differentiation may influence the later neuronal network formation. Therefore, it is desirable to develop a technique that enables us to stably maintain a transgene and to induce the expression of genes of interest by design.

Up to now, transposable elements, such as Sleeping Beauty, piggyBac and *Tol2*, have been utilized for integrating a gene of interest into the chromosome for steady expression [9]–[11]. For example, the *Tol2*-flanking regions are integrated into the chromosome in the presence of transposase. Furthermore, because the *Tol2* transposon, which originated in medaka fish, possesses an ability to undergo transposition in many kinds of species, including chick and mouse [12], it has been successfully applied to *in ovo* or *in utero* electroporation gene transfer in model animals for stable gene expression [13].

Although the tetracycline-controlled gene expression system, in which transcription is reversibly turned on or off in the presence of tetracycline, is one of the most frequently employed methods for inducible gene expression, we sometimes experienced a leakage in expression with this system in spite of the tetracycline control. A recent study showed that *Tol*2-mediated gene transfer systems with the tetracycline-induced expression system maintain controllable gene expression during chick embryogenesis [14]–[16]. However, it is unclear if the application of this system to induce the knockdown of endogenous genes with tight expression control at a particular point in mouse development can be expanded through the maturation period and into adulthood. Because siRNA is more stable than single-stranded RNA and siRNA within the RNA-induced Silencing Complex (RISC) are re-used after the degradation of the target mRNA, small amounts of siRNA are enough to cause knockdown effects [17], [18]. As a result, tightly controlled expression is crucial for the evaluation of the effects of gene knockdown. Therefore, generating tightly controlled inducible expression systems is pivotal to elucidate the *in vivo* function of a gene of interest using a knockdown approach without the influence of earlier events.

In this study, by combining the tightly controlled inducible gene expression cassette with the *Tol2* system, we developed a new tool that enables us to knockdown the gene of interest at any point during development.

## Results and Discussion

### Construction of a *Tol2* transposable vector for the inducible expression of the mir30-based knockdown cassette

**Figure 1:**
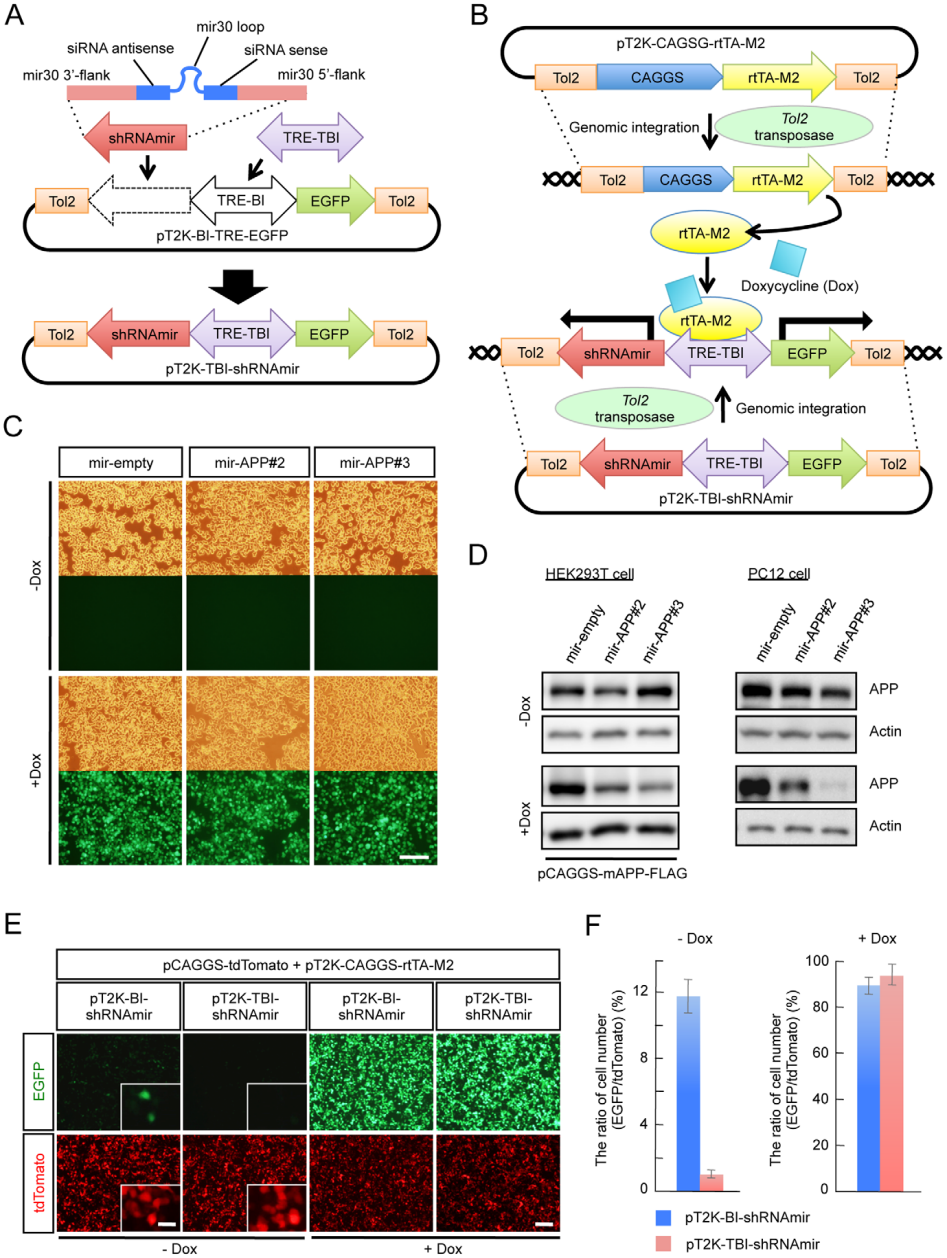
The *Tol2* transposable vector enables inducible knockdown from a stably integrated knockdown cassette. (A) A schematic diagram of the pT2K-TBI-shRNAmir vector. The shRNAmir cassette was inserted into the pT2K-BI-TRE-EGFP vector, and TRE-BI was replaced with TRE-TBI. The shRNAmir cassette consisted of the hairpin stem, which is composed of siRNA sense and antisense strands designed for the knockdown of the target gene, a loop derived from human mir30, and mir30 flanking sequences on the 3′ and 5′ sides of the hairpin. (B) A schematic diagram showing the principle of induction of knockdown from the genomically integrated shRNAmir cassette. The *Tol2*-flanked region of the plasmids were excised and integrated into the chromosome using *Tol2* transposase. In the presence of Doxycycline (Dox), rtTA-M2 bound to TRE-TBI, and the expression of both EGFP and the mir30-based knockdown cassette were induced under the control of TRE-TBI. (C) Expression of EGFP, induced from the each of pT2K-TBI-shRNAmir vectors (mir-empty, mir-APP#2 and mir-APP#3), was observed in almost all PC12 cells following Dox administration. The upper panels show the bright-field images. Scale bar, 100 µm. (D) Immunoblot analyses for evaluating the knockdown efficiency against APP. Actin was used as a loading control. (E) The basal expression (−Dox) and the induced expression (+Dox) of EGFP from the pT2K-BI-shRNAmir and pT2K-TBI-shRNAmir vectors in HEK293T cells. pCAGGS-tdTomato was co-transfected as a transfection control. Inset shows a higher magnification. Scale bars: 100 µm, inset 20 µm. (F) Ratio of the number of EGFP-positive cells to tdTomato-positive cells between the cells expressing pT2K-BI-shRNAmir and pT2K-TBI-shRNAmir with or without Dox. (mean ± SEM, n = 3). Abbreviations of the vector name and their components are listed in the table (Table S1).

For the purpose of conditional gene knockdown at any point during development, we constructed the inducible knockdown vector pT2K-TBI-shRNAmir based on the *Tol2* transposable vector pT2K-BI-TRE-EGFP by inserting the mir30 cassette (shRNAmir) and exchanging the promoter (Figure 1A). pT2K-BI-TRE-EGFP is originally developed for the stable integration and conditional expression of transgenes in chick embryos [13], which can be used to express EGFP and a gene of interest under the control of a bidirectional tetracycline-responsive promoter that consists of the tetracycline-responsive element (TRE-BI) between two minimal cytomegalovirus (CMV) promoters. For better control of inducible expression, we first replaced TRE-BI with TRE-Tight-BI (TRE-TBI), which has a modified TRE that yields high induction levels and low basal promoter activity compared with TRE [19]. The shRNAmir cassette has a hairpin stem composed of siRNA sense and antisense strands designed to knockdown the target gene, a loop derived from human mir30 and mir30 flanking sequences on both sides of the hairpin [20], [21]. By adopting this mir30-based shRNA expression cassette, conditional shRNA expression from an RNA polymerase II promoter, such as CMV, can be achieved.

pT2K-CAGGS-rtTA-M2, which expresses the modified reverse tetracycline-controlled transactivator (rtTA-M2) [22], and pT2K-TBI-shRNAmir are designed such that *Tol2*-flanking regions are integrated into the chromosome in the presence of *Tol2* transposase. The rtTA-M2 specifically binds to TRE-TBI in the presence of Doxycycline (Dox), an analogue of tetracycline, and activates transcription (Figure 1B). Thereby, shRNAmir is induced simultaneously with EGFP from the *Tol2*-flanking region of the pT2K-TBI-shRNAmir vector, which is integrated into the chromosome, with tightly controlled expression.

We designed the knockdown cassette based on the miRNA backbone for pT2K-TBI-shRNAmir. There are some advantages in using the miRNA-based knockdown cassette: (1) Compared with the conventional stem-loop-based shRNAs, miRNA-based hairpins have been proven to exhibit lower cellular toxicity effects [23], [24]. (2) In addition, because the miRNA based cassette is transcribed by RNA polymerase II, tissue or cell type specific expression of the knockdown can be achieved with an appropriate promoter. It has been reported that, while it is not inducible nor an miRNA-based knockdown system, the combination of the *Tol2* system with glial lineage-specific promoters drives the glial cell-specific expression of transgenes in the mouse cerebral cortex when transfected using *in utero* electroporation gene transfer [25]. Because a large number of mouse lines in which Cre recombinase is expressed in a tissue or a cell type-specific manner have been reported and are available [26], it is likely that the use of these bioresources with specific promoters expands the usefulness of our inducible knockdown system. For instance, this approach enables knockdown in a specific cortical layer or in a specific neuronal subtype.

### Verification of knockdown effect against an endogenous target gene by shRNAmir expressed from a stably integrated knockdown cassette

We examined whether the pT2K-TBI-shRNAmir vectors were integrated into the chromosomes. If the vectors were not integrated into the chromosome, these vectors were re-distributed into two daughter cells after mitosis. Eventually the number of these vectors in individual cells should become very small. In contrast, in the cells harboring such vectors in their chromosomes, these vectors should be retained after cell division. We tested the ability of this vector to integrate into the genome with APP as an inducible knockdown target. We used pT2K-TBI-shRNAmir vector as a control empty vector (mir-empty), and designed two pT2K-TBI-shRNAmir constructs, pT2K-TBI-shRNAmir-APP#2 (mir-APP#2) and pT2K-TBI-shRNAmir-APP#3 (mir-APP#3), which targeted different regions of the APP sequence.

We transfected mir-empty, mir-APP#2 or mir-APP#3 into PC12 cells, which express APP endogenously, together with pT2K-rtTA-M2, pCAGGS-T2TP (*Tol2* transposase) and pCAGGS-tdTomato for the visualization of transfected cells. The cells were cloned and then subcultured every 4 to 5 days for 47 days until the expression of the transfection control, tdTomato, became undetectable. Even after 47 days of culturing, almost all of the cells still expressed EGFP upon induction by Dox, confirming that these vectors were stably integrated into the genome (Figure 1C).

We next determined whether mir-APP#2 and mir-APP#3 knocked down APP. Transient expression of either mir-APP#2 or mir-APP#3 resulted in the reduction of APP in the presence of Dox in HEK293T cells exogenously expressing APP following transfection with pCAGGS-mAPP-FLAG. A stronger knockdown effect was observed with mir-APP#3 (Figure 1D). Furthermore, we examined the efficiency of knockdown induced by Dox in PC12 cells in which the APP knockdown vectors had been integrated into the genome. In the presence of Dox, APP was knocked down suggesting that the chromosome-integrated pT2K-TBI-shRNAmir vector was able to induce the knockdown of the target gene. Consistent with the results in HEK293T cells, mir-APP#3 gave a stronger activity of knockdown against APP (Figure 1D). Tight control of the expression of the pT2K-TBI-shRNAmir vector was also verified in HEK293T cells. The pT2K-TBI-shRNAmir vector showed an approximate 12-fold reduction in leakage compared with the pT2K-BI-shRNAmir vector in the absence of Dox (Figs. 1E and 1F). Therefore, changing promoters from TRE-BI to TRE-TBI contributes to the reduction of undesired knockdown effects due to leakage of the expression without induction.

### The pT2K-TBI-shRNAmir vector is integrated into the genome, and its inducible expression is tightly controlled in the mouse cortex

**Figure 2:**
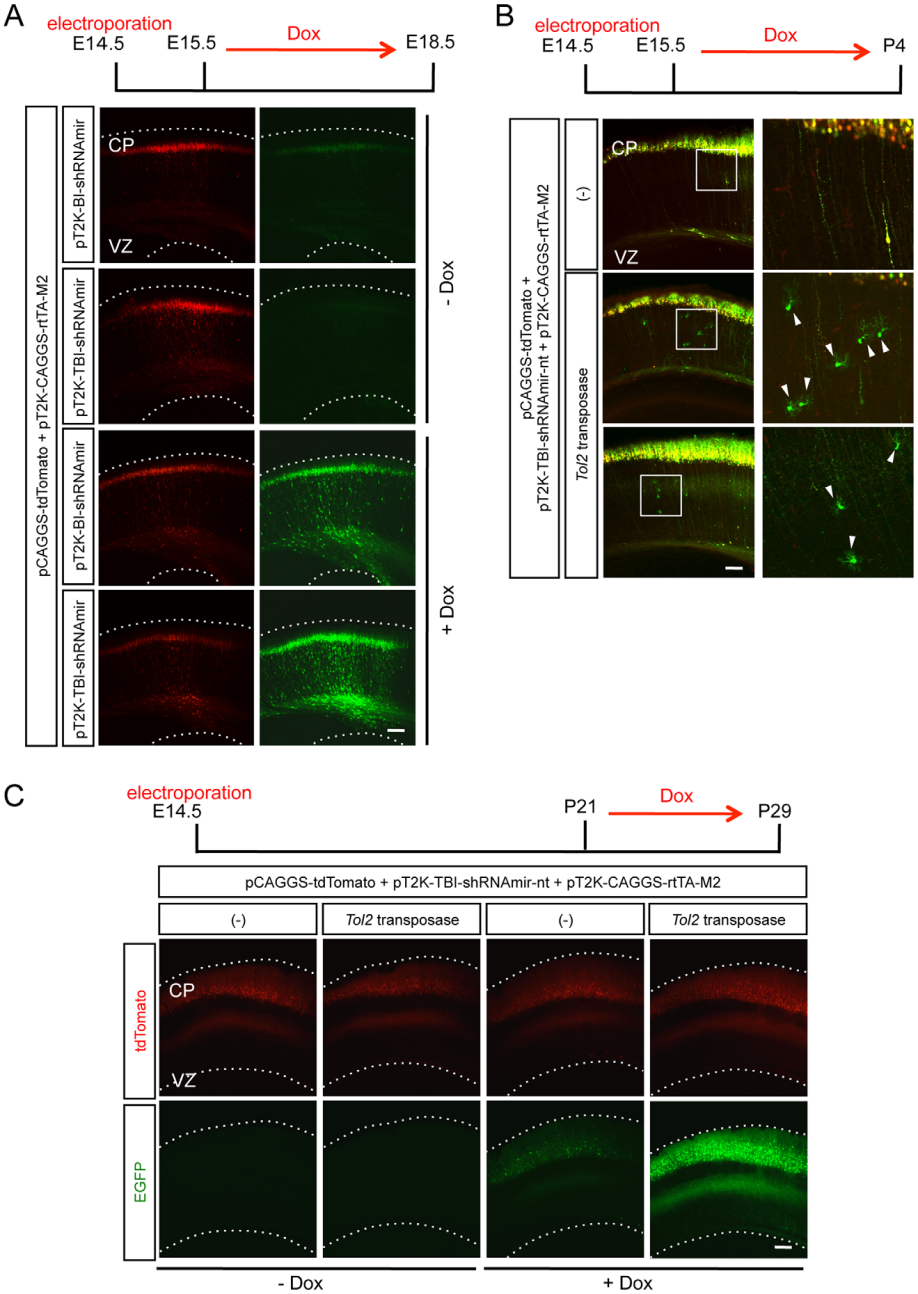
*Tol2* knockdown vectors were genomically integrated and the inducible expression was tightly controlled *in vivo*. (A) Representative neocortical sections showing the basal expression and induced expression of EGFP derived from the pT2K-BI-shRNAmir or pT2K-TBI-shRNAmir vectors. (B) The retention and expression of inducible knockdown vector in glial cells. In the presence of *Tol2* transposase, EGFP was observed in the glial cells (arrowheads). The right panels are higher magnification views of the boxed regions in the left panels. (C) Representative neocortical sections showing the retention and expression of the inducible knockdown vector in the adult cortex. CP, cortical plate; VZ, ventricular zone. Scale bars, 100 µm.

Next, we asked whether our *Tol2* inducible knockdown vector, pT2K-TBI-shRNAmir, works *in vivo*, especially in the developing cerebral cortex in mice. First, we tested whether the expression from our pT2K-TBI-shRNAmir was tightly regulated. pT2K-BI-shRNAmir or pT2K-TBI-shRNAmir was electroporated into mouse cerebral cortices together with pT2K-CAGGS-rtTA-M2 and pCAGGS-tdTomato, and gene expression was then induced by Dox administration. For both pT2K-BI-shRNAmir and pT2K-TBI-shRNAmir, the expression of EGFP that had been induced simultaneously with the shRNAmir knockdown cassette from the bidirectional promoter was observed following Dox administration. However, we detected much weaker EGFP expression from pT2K-TBI-shRNAmir without induction than that from pT2K-BI-shRNAmir (Figure 2A). These results indicated that the expression of pT2K-TBI-shRNAmir was controlled tightly *in vivo*, similar to our *in vitro* results.

Next, we asked whether pT2K-TBI-shRNAmir was integrated into the genome of the developing cortex in mice. If the *Tol2*-flanked regions were integrated into the genome by the *Tol2* transposase, EGFP expression would be observed even in the glial cells in which transfected non-integrated vectors were otherwise diluted due to cell proliferation. pT2K-TBI-shRNAmir-non-target (pT2K-TBI-shRNAmir-nt), pT2K-CAGGS-rtTA-M2 and pCAGGS-tdTomato were electroporated into the mouse cerebral cortex with or without the *Tol2* transposase expression vector, and gene expression was induced by Dox at E15.5. Cortices were observed at P4, by which point glial cells must have experienced multiple rounds of cell divisions (Figure 2B). At P4, EGFP expression was clearly confirmed in the glial cells with *Tol2* transposase but not without *Tol2* transposase (Figure 2B). These results showed that *Tol2* knockdown vectors were integrated into the genome in the presence of *Tol2* transposase. In addition, we assessed whether the knockdown cassette is stably maintained throughout development by observing the expression of EGFP, which is under the control of the bidirectional promoter TRE-TBI, after 4 weeks of postnatal life with Dox administration for a week before the harvest. We did not detect any obvious leakage of EGFP expression in the adult mouse cortex without induction. In Dox-treated animals, a drastic induction of EGFP expression was observed in the cortices that had been electroporated with the transposase expression vector (Figure 2C).

Although plasmids were introduced into the neural progenitors in the VZ by *in utero* electroporation gene transfer, these progenitors, as well as recently recognized intermediate progenitors in the subventricular zone [27], undergo mitosis before finally settling into the cortical plate. Because an episomally located vector is likely to be diluted during mitosis, whereas a transposed vector is not, it is probable that higher inducible expression was observed in the cortical neurons of the adult brain when the *Tol2* transposase vector was co-transfected (Figure 2C). Indeed, EGFP expression was weaker in superficial neurons, which are likely to experience more mitotic cycles, compared with those in deeper layers in the absence of *Tol2* transposase activity (Figure 2C, 3^rd^ column).

### Inducible knockdown of a gene that is essential for the cortical development impairs radial migration

**Figure 3:**
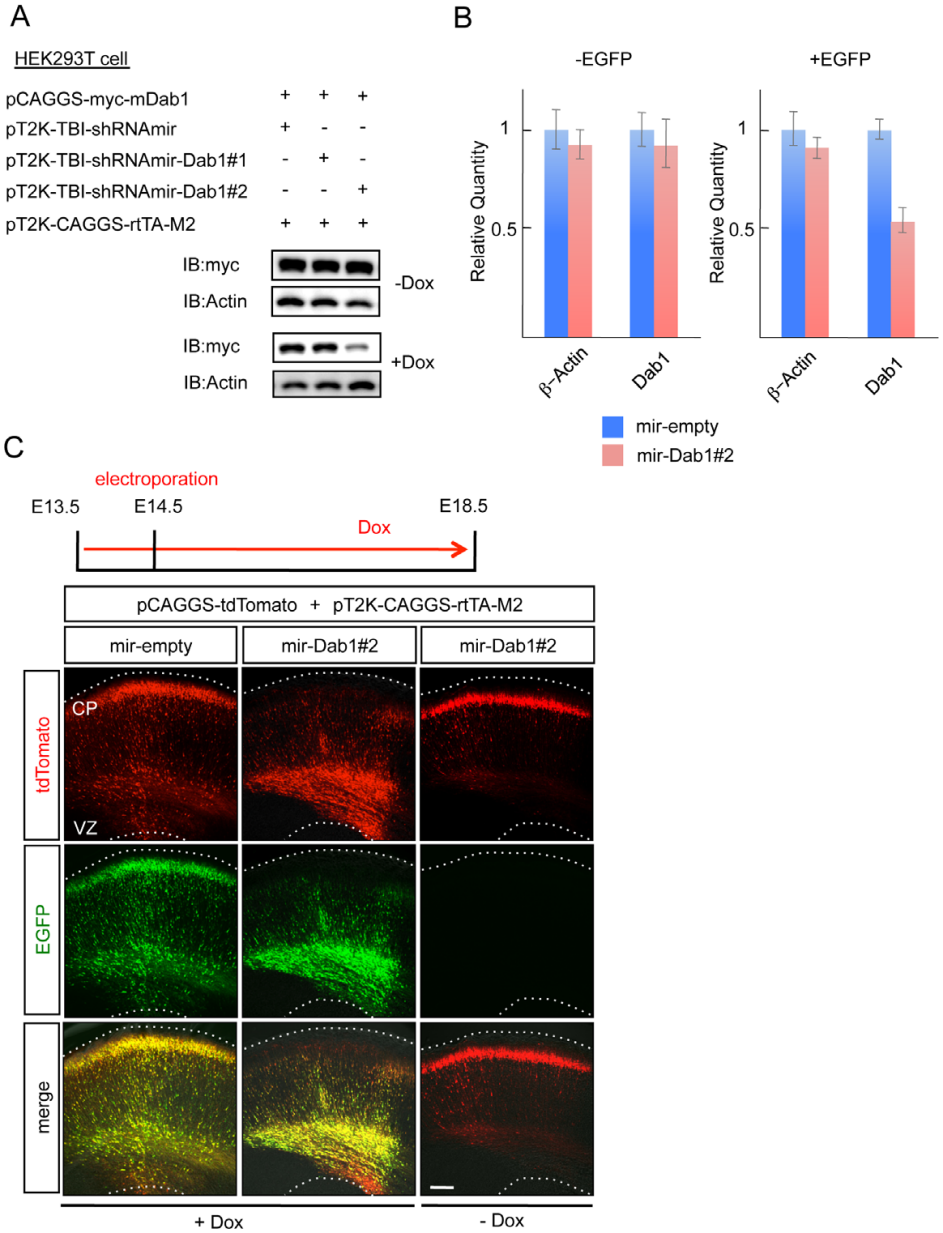
Inducible knockdown of a gene essential for cortical development impaired radial migration. (A) Immunoblot analysis of the inducible knockdown against the exogenously expressed Dab1 in HEK293T cells. Dab1 was decreased in the presence of the *Tol2*-inducible knockdown vector pT2K-TBI-shRNAmir-Dab1#2 following the application of Dox. (B) Quantitative real-time PCR data showing the efficiency of inducible knockdown against the endogenous Dab1 in cortical neurons. In the EGFP-positive cells that had been electroporated with pT2K-TBI-shRNAmir-Dab1#2, the expression level of Dab1 was significantly decreased compared with the control. (C) Inducible knockdown of Dab1 during cortical development. The empty control vector did not inhibit neuronal migration. The induction of mir-Dab1#2 expression resulted in impaired radial migration in the presence of Dox but not without Dox. Scale bar, 100 µm.

Although our observations showed that the combination of the *Tol2* inducible knockdown vector and the *in utero* electroporation gene transfer method enables the induction of gene expression at the time of interest, it remains unclear whether the inducible knockdown is effective *in vivo*. We performed inducible knockdown against *Dab1*, a molecule that plays an important role in radial migration under the Reelin signaling pathway, in addition to APP (Figure S1). First, the efficiency of the inducible knockdown against the exogenously expressed Dab1 was examined in HEK293T cells. Because pT2K-TBI-shRNAmir-Dab1#2 showed a knockdown effect in a Dox-dependent manner, subsequent experiments were performed with this vector (Figure 3A). To reveal the knockdown efficiency against the endogenous Dab1 in the cerebral cortex, we performed *in utero* electroporation in the mouse cortex. Knockdown was then induced by the administration of Dox. Because the knockdown cassette expressed EGFP from the bidirectional promoter, EGFP-expressed cells were FACS-sorted, and quantitative real-time PCR was performed. In the EGFP-positive cells, Dab1 expression was decreased significantly compared with control cells (Figure 3B). It has been reported that reduction of the Dab1 protein in the cerebral cortex results in the malformation of the cortical lamination [8], [28]. Therefore, we assessed the effect of the induction of *Dab1* knockdown and the leakage of this knockdown cassette in the absence of Dox. Radial migration was inhibited in the cortex following the induction of the *Dab1* knockdown, but not in brains exposed to the control vector or in the absence of Dox (Figure 3C). These results indicated that the *Tol2* inducible knockdown vector was tightly controlled and effectively knocked down the expression of the endogenous gene without expression leakage.

Molecules that control neuronal migration, including regulators of the cytoskeleton and cell adhesion molecules [29], [30], also affect other processes, such as axon projection and synaptogenesis [31], [32], after the cessation of migration. Application of the *Tol2* inducible knockdown system described here allows us to investigate gene functions in each event separately by controlling the timing of the gene silencing.

Currently, the demand for the development of tools for conditional genetic manipulation, especially in a small number of neurons, is increasing. Young *et al.* create one such method, SLICK (single-neuron labeling with inducible Cre-mediated knockout) that utilizes drug inducible Cre-mediated knockout technology [33]. With SLICK, they can visualize a whole neuronal shape by co-expressing YFP (yellow fluorescent protein) together with Cre, which is also accomplished by our system with EGFP. However, they knockout a gene of interest in an irreversible manner with SLICK, whereas we knockdown a gene of interest in a reversible manner, in which we can bring back the gene expression later. Moreover, specific Cre-expressing neurons are targeted in SLICK, whereas our system enables us to knockdown a gene in any electroporated neurons. To facilitate functional genomics in the nervous system, an appropriate methodology should be taken for conditional genetic manipulation to the purpose. Our system has the potential to be a tool with which to elucidate the *in vivo* molecular mechanisms of later stages of development.

## Materials and Methods

### Animals

ICR mice were purchased from SLC (Hamamatsu, Japan). Noon on the day when the presence of a vaginal plug was confirmed was designated as embryonic day 0.5 (E0.5). For pups, the day of birth was designated as postnatal day 0 (P0). All experiments were conducted in accordance with the guidelines for animal experiments of the University of Fukui, and all efforts were made to minimize both the number of animals used and their suffering. The protocol was approved by the Committee on the Ethics of Animal Experiments of the University of Fukui (Permit Number: 23012).

### Vector constructions

Mouse full-length Dab1 (GenBank accession number NM_177259) cDNA was cloned from E14.5 mouse brains by the polymerase chain reaction (PCR). For pCAGGS-myc-mDab1, the Dab1 cDNA was amplified by PCR with the primers 5′-AACCTCGAGATGTCAACTGAGACAGAACTTCAAG-3′ and 5′-GCTCTCGAGCTAGCTACCGTCTTGTGGACTTATATTATC-3′. The PCR product was cloned into the XhoI sites of the pCAGGS-myc vector. Mouse full-length APP (GenBank accession number NM_007471) cDNA was cloned from E14.5 mouse brains by PCR. For pCAGGS-mAPP-FLAG, the APP cDNA was amplified by PCR with the primers 5′-AATAGATATCATGCTGCCCAGCTTGG-3′ and 5′- GAAGATATCGTTCTGCATTTGCTCAAAGAAC-3′. The PCR product was cloned into the EcoRV sites of the pCAGGS-FLAG-IRES-GFP vector. For the construction of pT2K-BI-shRNAmir, the mir-30 cassette was excised from pCAG-mir30 (Addgene plasmid 14758; Addgene, Cambridge, MA) [34] with XbaI/HindIII sites and inserted into the EcoRV/ApaI site of pT2K-BI-TRE-EGFP [13] with blunt-end ligation. The EcoRI sites flanking the TRE sequence were then eliminated. For the construction of pT2K-TBI-shRNAmir, the TRE-BI sequence was removed from pT2K-BI-shRNAmir using the BamHI/NcoI site, then blunted and self-ligated to reform the BamHI site. The TRE-Tight-BI sequence was excised with BamHI/BglII from pTRE-Tight-BI (Clontech, Palo Alto, CA), which lacks the EcoRI and XhoI sites, and then inserted into the BamHI site of pT2K-BI-shRNAmir.

### RNA interference

The mir30-based shRNA was synthesized by PCR with the primers 5′-CAGAAGGCTCGAGAAGGTATATTGCTGTTGACAGTGAGCG-3′ and 5′-CTAAAGTAGCCCCTTGAATTCCGAGGCAGTAGGCA-3′ using the following template oligonucleotides: for shRNAmir-non-target (shRNAmir-nt), 5′-TGCTGTTGACAGTGAGCGATCTCGCTTGGGCGAGAGTAAGTAGTGAAGCCACAGATGTACTTACTCTCGCCCAAGCGAGAGTGCCTACTGCCTCGGA-3′; for shRNAmir-APP#2, 5′-TGCTGTTGACAGTGAGCGCGCACTAACTTGCACGACTATGTAGTGAAGCCACAGATGTACATAGTCGTGCAAGTTAGTGCTTGCCTACTGCCTCGGA-3′; for shRNAmir-APP#3, 5′-TGCTGTTGACAGTGAGCGCGCTGACAAGAAGGCCGTTATCTAGTGAAGCCACAGATGTAGATAACGGCCTTCTTGTCAGCTTGCCTACTGCCTCGGA-3′; for shRNAmir-Dab1#1, 5′-TGCTGTTGACAGTGAGCGCCCTCCTCCTCTTAGCACTAAATAGTGAAGCCACAGATGTATTTAGTGCTAAGAGGAGGAGGATGCCTACTGCCTCGGA-3′; for shRNAmir-Dab1#2, 5′-TGCTGTTGACAGTGAGCGCAAGGATAAGCAGTGTGAACAATAGTGAAGCCACAGATGTATTGTTCACACTGCTTATCCTTTTGCCTACTGCCTCGGA-3′. The PCR product was cloned into the XhoI/EcoRI sites of the pT2K-TBI-shRNAmir vector. The underlined sequences correspond to the sense and antisense target sequences, respectively.

### Cell culture, Transfection and Immunoblotting analysis

Cells were cultured in Dulbecco's modified Eagle's medium (DMEM) supplemented with either 10% fetal bovine serum for HEK293T cells (ATCC No. CRL-2828) or 10% fetal bovine serum and 5% horse serum for PC12 cells (ATCC No. CRL-1721). Plasmid DNAs were transfected into cells using the Lipofectamine™ 2000 transfection reagent (Invitrogen, Carlsbad, CA). The cells were lysed with the lysis buffer (20 mM HEPES-NaOH, pH 7.5, 100 mM NaCl, 3 mM MgCl_2_, 1 mM dithiothreitol, 1 mM EGTA, 0.5% Nonidet P-40) supplemented with 1% Protease Inhibitor Cocktail (Sigma, St. Louis, MO). After centrifugation, the supernatant was mixed with Laemmli sample buffer, boiled, and subsequently separated on SDS-polyacrylamide gels. The separated proteins were transferred to polyvinylidene difluoride membranes. After blocking for 30 min in 5% skim milk-TBST (50 mM Tris-HCl, pH 7.4, 150 mM NaCl, 0.1% Tween 20), the membranes were probed for 1 h with the primary antibodies in 5% skim milk-TBST. The membranes were then washed three times with TBST and incubated with the secondary antibodies coupled to horseradish peroxidase. Immunoblotting was visualized using an Immobilon Western Chemiluminescent HRP Substrate (Millipore, Bedford, MA) on a LAS-3000 mini imaging system (Fujifilm, Tokyo, Japan). The following antibodies (and dilutions) were used: the mouse anti-myc antibody (1∶1000) (Santa Cruz Biotechnology, Santa Cruz, CA), anti-FLAG antibody (1∶1000) (Sigma, St. Louis, MO), anti-actin antibody (1∶500) (Sigma, St. Louis, MO) and the mouse anti-APP antibody (1∶1000) (Millipore, Bedford, MA).

### Semi-quantitative analysis

For the semi-quantitative analysis of the ratio of EGFP-positive cells to tdTomato-positive cells, images were obtained with a fluorescence microscope (Axio observer A1; Carl-Zeiss, Oberkochen, Germany), and the number of EGFP-positive and tdTomato-positive cells were counted using NIH Image J software.

### *In utero* electroporation

Plasmids were transfected by *in utero* electroporation using previously described methods with some modifications [4], [5], [35]. Briefly, on E14.5, pregnant female ICR mice were anaesthetized with an intraperitoneal injection of 2,2,2-tribromoethanol (200–300 mg/kg body weight) before the experiments. Approximately 1–2 µl of plasmid solution (1–5 µg/µl) was injected into the lateral ventricle of the embryos. Relative ratio of plasmids in mixed solution is as follows. pCAGGS-tdTomato: pT2K-CAGGS-rtTA-M2: pT2K-BI-shRNAmir or pT2K-TBI-shRNAmir: pCAGGS-T2TP (*Tol2* transposase) = 1∶5∶10∶10. After soaking the uterine horn with PBS, the head of the embryo was pinched with a forceps-type electrode (CUY650P3 for E12.5 or CUY650P5 for E14.5; Nepa gene, Chiba, Japan), and five cycles of square electric pulses (35 V, 50 ms for E12.5 or 45 V, 50 ms for E14.5) at intervals of 950 ms were delivered using an electroporator (CUY21EDIT; Nepa Gene, Chiba, Japan). For the analysis of inducible expression in the cerebral cortex, the brains were fixed with 4% paraformaldehyde in 0.1 M phosphate buffer, pH 7.4, cut coronally into 100 µm slices with a Vibratome (DTK-1000; DSK, Kyoto, Japan), and imaged on a laser-scanning confocal microscope (LSM 5 PASCAL; Carl-Zeiss, Oberkochen, Germany).

### FACS sorting and quantitative real-time PCR

pT2K-TBI-shRNAmir or pT2K-TBI-shRNAmir-Dab1#2 was electroporated with pT2K-CAGGS-rtTA-M2 into the mouse cerebral cortex at E14.5. The cells were collected at E18.5 using FACS (FACS Aria II, BD Bioscience, San Jose, CA). Doxycycline (Dox) was administrated to mice at 2 mg/ml in a 5% sucrose solution in drinking water during the experiment from E14.5 to E18.5. To make cDNA for real-time PCR from the total RNA of FACS-sorted cells, reverse transcription was performed using reverse transcriptase (ReverTra Ace™; Toyobo, Osaka, Japan). Real-time PCR reactions were performed on an ABI StepOnePlus™ Real-Time PCR System (Applied Biosystems, Foster city, CA) using the SYBR Green PCR Master Mix (Thunderbird™; Toyobo, Osaka, Japan). The thermal cycling was performed using the following cycle conditions: the initial denaturation step at 95°C for 60 s, followed by 40 cycles at 95°C for 15 s, and 60°C for 60 s. The experiments were carried out in triplicate. The relative quantification of gene expression compared to a control was determined using the ΔΔCt method. Changes in gene expression were normalized to the internal control gene PRS18. The following gene-specific primers were used for real-time PCR analysis: for PRS18, 5′-AACGGTCTAGACAACAAGCTG-3′ and 5′-AGTGGTCTTGGTGTGCTGAC-3; for β-actin, 5′-ATGCTCCCCGGGCTGTAT-3′ and 5′-CATAGGAGTCCTTCTGACCCATTC-3; and for Dab1, 5′-AGCTCAAGGGTGTTGTTGCT-3′ and 5′- TGTGATGTCCTTCGCAATGT-3.

## Supporting Information

Figure S1 **Knockdown effects of shRNA- and miRNA-based knockdown vectors against APP on the radial migration using the various promoters.** APP was knocked down using the shRNA- or miRNA-based knockdown vectors (Text S1) in the cerebral cortex. (A) Immunoblot analyses of the knockdown against the exogenously expressed mouse APP in HEK293T cells. (B) Mouse cortices were electroporated with vectors at E14.5 and observed at E18.5. Only the knockdown vector with mU6pro-shAPP#2 inhibited neuronal migration. (C) Immunoblot analyses of the knockdown efficiency against the exogenously expressed mouse APP in HEK293T cells using shRNAmir vector with various promoters. (D) CMV-driven or mU6-driven knockdown vectors were transfected at E14.5 and cortices were observed at E18.5. There was no significant change. Scale bars, 100 µm.
(TIF)

Table S1 **The nomenclature of the vectors and components.**
(PDF)

Text S1 **Supporting methods for vector constructions.**
(DOC)
