# Supplementary figures and images for: A Tightly Controlled Conditional Knockdown System Using the Tol2 Transposon-Mediated Technique
Source: PLoS One. 2012 Mar 13;7(3):e33380. doi: 10.1371/journal.pone.0033380 (PMC3302819; doi:10.1371/journal.pone.0033380)

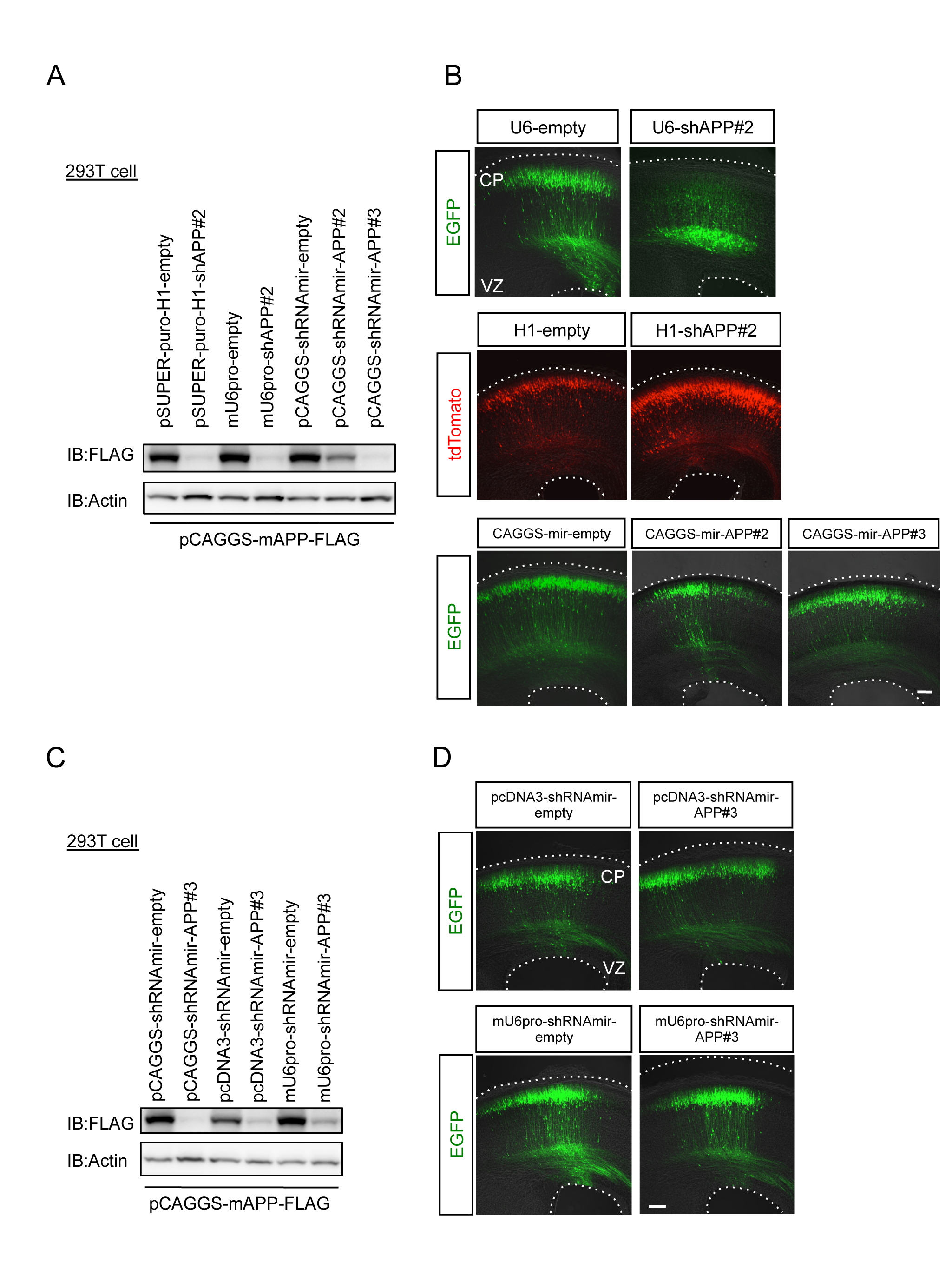

Supplement: Figure S1 — Knockdown effects of shRNA- and miRNA-based knockdown vectors against APP on the radial migration using the various promoters. APP was knocked down using the shRNA- or miRNA-based knockdown vectors (Text S1) in the cerebral cortex. (A) Immunoblot analyses of the knockdown against the exogenously expressed mouse APP in HEK293T cells. (B) Mouse cortices were electroporated with vectors at E14.5 and observed at E18.5. Only the knockdown vector with mU6pro-shAPP#2 inhibited neuronal migration. (C) Immunoblot analyses of the knockdown efficiency against the exogenously expressed mouse APP in HEK293T cells using shRNAmir vector with various promoters. (D) CMV-driven or mU6-driven knockdown vectors were transfected at E14.5 and cortices were observed at E18.5. There was no significant change. Scale bars, 100 µm. (TIF) [file pone.0033380.s001.tif]
