# Supplementary material for: A Tightly Controlled Conditional Knockdown System Using the Tol2 Transposon-Mediated Technique
Source: PLoS One. 2012 Mar 13;7(3):e33380. doi: 10.1371/journal.pone.0033380 (PMC3302819; doi:10.1371/journal.pone.0033380)
Supplement: Table S1 — The nomenclature of the vectors and components. (PDF) [file pone.0033380.s002.pdf]

Table S1. The nomenclature of the vectors and components

	Abbreviation	Full name	Figure
vector	*Tol2*-transposase	pCAGGS-T2TP	1B, 2B, 2C
	mir-empty	pT2K-TBI-shRNAmir	1C, D, 3B, C
	mir-APP#2	pT2K-TBI-shRNAmir-APP#2	1C, D
	mir-APP#3	pT2K-TBI-shRNAmir-APP#3	1C, D
	mir-Dab1#2	pT2K-TBI-shRNAmir-Dab1#2	3B,C
	H1-empty	pSUPER-puro-H1-empty	S1B
	H1-shAPP#2	pSUPER-puro-H1-shAPP#2	S1B
	U6-empty	mU6pro-empty	S1B
	U6-shAPP#2	mU6pro-shAPP#2	S1B
	CAGGS-mir-empty	pCAGGS-shRNAmir-empty	S1B
	CAGGS-mir-APP#2	pCAGGS-shRNAmir-APP#2	S1B
	CAGGS-mir-APP#3	pCAGGS-shRNAmir-APP#3	S1B
vector component	shRNAmir	mir30-based knockdown cassette	1A, B
	TRE-BI	bidirectional tetracycline-responsive element	1A
	TRE-TBI	tightly controled bidirectional tetracycline-responsive element	1A, B
	Tol2	Tol2 transposable element	1A, B
	EGFP	enhanced green fluorescent protein	1A, B
	rtTA-M2	modified reverse tetracycline-controlled transactivator	1B
others	Dox	Doxycycline	1C,D,E,F, 2A,B,C, 3A,C

'Figure' column indicates figure panels in which abbreviations are appeared.
