# Supplementary material for: A Tightly Controlled Conditional Knockdown System Using the Tol2 Transposon-Mediated Technique
Source: PLoS One. 2012 Mar 13;7(3):e33380. doi: 10.1371/journal.pone.0033380 (PMC3302819; doi:10.1371/journal.pone.0033380)
Supplement: Text S1 — Supporting methods for vector constructions. (DOC) [file pone.0033380.s003.doc]

**Text S1. Supporting methods for vector constructions.**

Constructs of short hairpin RNAs (shRNAs) were prepared in the mouse U6 shRNA promoter (mU6pro) vector, which has a mouse U6 RNA polymerase III promoter [1], or in the pSUPER RNAi system with the H1 RNA polymerase III promoter (Oligoengine, Seattle, WA, USA). The oligonucleotide sequences used in the construction of the vector were as follows: for mU6pro-shAPP#2, 5'-TTTGGCACTAACTAGCACGACTATGGACAAGAGACATAGTCGTGCAAGTTAGTGCTTTTT-3' and 5'-CTAGAAAAAGCACTAACTTGCACGACTATGTCTCTTGTCCATAGTCGTGCTAGTTAGTGC-3'; for pSUPER-puro-H1-shAPP#2, 5'-GATCCCCGCACTAACTAGCACGACTATGTTCAAGAGACATAGTCGTGCAAGTTAGTGCT-3' and 5'-AGCTTAAAAAGCACTAACTTGCACGACTATGTCTCTTGAACATAGTCGTGCTAGTTAGTGCGGG-3'. The oligonucleotides were annealed and then ligated into BbsI/XbaI sites of the mU6pro vector and BglIII/HindIII sites of the pSUPER-puro-H1 (Oligoengine, Seattle, WA, USA). The underlined sequences correspond to the sense and antisense target sequences, respectively. For the mir30-based shRNA vector with CMV promoter; pcDNA3-shRNAmir-empty and pcDNA3-shRNAmir-APP#3, and U6 promoter; mU6pro-shRNAmir-empty and mU6pro-shRNAmir -APP#3, knockdown cassette was amplified by PCR from pCAGGS-shRNAmir and pCAGGS-shRNAmir-APP#3 with the primers 5'-TAATGGATCCTCGACTAGGGATAACAGGG-3' and 5'-ATGCTCTAGAAAAAAAGTGATTTAATTTATACCATTTTAATTC-3'. Then, the PCR product was cloned into the BamHI/XbaI sites of the pcDNA3 vector and mU6pro vector.

**References**

1. Yu J-Y, DeRuiter SL, Turner DL (2002) RNA interference by expression of short-interfering RNAs and hairpin RNAs in mammalian cells. Proc Natl Acad Sci U S A 99: 6047–6052.
